# How membrane receptors tread the fine balance between symbiosis and immunity signaling

**DOI:** 10.1073/pnas.2106567118

**Published:** 2021-06-03

**Authors:** Chai Hao Chiu, Uta Paszkowski

**Affiliations:** ^a^Crop Science Centre, Department of Plant Sciences, University of Cambridge, Cambridge CB3 0LE, United Kingdom

Throughout their life cycle, plants have to respond appropriately to diverse microorganisms. While living alongside harmless commensals and warding off disease-causing and nutrient-seeking pathogens, plants also engage in intimate endosymbiosis with microorganisms that deliver scarce mineral nutrients. In particular, the mutually beneficial symbiosis with arbuscular mycorrhizal fungi (AMF) is prevalent and is thought to have been instrumental for plant colonization of the terrestrial landscape ca. 450 million years ago. Until today, it remains important for plant nutrition (1). The molecular mechanisms that underlie the decisions made by plants to engage with the appropriate microorganisms are an area of intense research, and knowledge gleaned could enable the development of crops that benefit more from and are compromised less by their microbiomes.

One of the earliest steps of microbe recognition by plants involves the perception of conserved molecules not found in plants, also known as microbe-associated molecular patterns (MAMPs). Over two decades, plant receptors have been identified that recognize their cognate MAMPs and transduce cellular signaling. These receptors recognize conserved MAMPs ranging from peptides (e.g., flagellin epitopes) to carbohydrates (e.g., microbial cell wall components), typifying the plants’ innate immune surveillance system (2). Perception of carbohydrate MAMPs such as chitin or peptidoglycan involve lysin motif receptor-like proteins (RLPs) or receptor-like kinases (RLKs) on the plasma membrane. The lysin motif, first identified in bacteriophages, is a carbohydrate-binding domain with affinity for polymers of *N*-acetylglucosamine (GlcNAc) (3), which forms chitin and is an integral part of peptidoglycan, and rhizobial nod factors, also known as lipo-chitooligosaccharides (LCOs). While long-chain chitooligosaccharides (COs) such as chitooctaose (CO8) and peptidoglycan are classically regarded as pathogen-associated, short-chain chitotetraose (CO4) and LCOs are symbiont-associated MAMPs—it is these MAMPs that are perceived by plants when engaging with both pathogens and symbionts.

Evidence from various plant species has implicated the role of LysM-RLKs/RLPs in carbohydrate perception for both immunity and symbiotic contexts. LysM-RLKs *Nod Factor Receptor 1* (*NFR1*) and *NFR5* were identified from a forward mutant screen in *Lotus japonicus* to be necessary for the perception of LCO (also known as nod factors) to initiate signaling in nitrogen-fixing symbiosis with rhizobia bacteria (4). In rice, affinity labeling established Chitin Elicitor Binding Protein (CEBiP) as the major CO-binding receptor (5). CEBiP and its coreceptor, Chitin Elicitor Receptor Kinase1 (CERK1), are required for chitin-triggered immunity (6).

The intriguing observation that rice *cerk1* mutants display reduced arbuscular mycorrhizal symbiosis suggested that the perception of pathogen-derived signals and symbiosis-promoting factors (also known as *myc factors* in analogy to rhizobial *nod factors*) involves a shared coreceptor (6). In fact, we now know that CERK1 is a major coreceptor in carbohydrate MAMP perception and is involved in both immunity and mycorrhizal symbiosis signaling in most [but not all (7)] of the plant species tested to date (8).

**Fig. 1.**
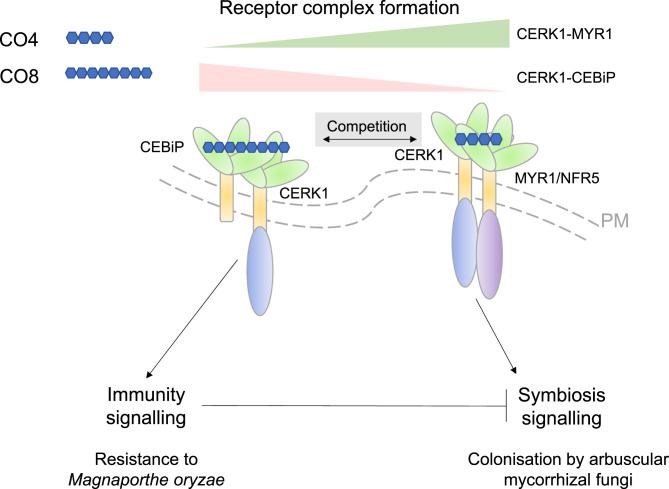
Summary of the findings and model of Zhang et al. (9). Zhang et al. provide evidence for receptor competition that allow rice plants to discriminate chitin signals and balance immunity and symbiosis signaling. In the presence of CO4, CERK1–CEBiP complex is reduced in favor of CERK1–MYR1 complex, activating the respective immune responses and symbiosis-associated nuclear calcium-spiking responses downstream of the respective receptor complexes. Although chito-octaose (CO8) activates nuclear calcium oscillations, it however had a weak suppressive effect on symbiosis development. CEBiP, Chitin Elicitor Binding Protein; CERK1, Chitin Elicitor Receptor Kinase1; CO4, short-chain chitin oligomers; CO8, long-chain chitin oligomers; MYR1, Myc Factor Receptor1; NFR5, Nod Factor Receptor5 (both MYR1 and NFR5 refer to the same gene in rice); PM, plasma membrane.

An attractive model for how plants discriminate mutualists from pathogens was, therefore, that the formation of different CERK1–coreceptor complexes results in different signaling outputs (6, 8). In PNAS, Zhang et al. (9) now provide evidence that, depending on the receptors that recruit CERK1, rice plants discriminate symbiotic versus immunity signals, as presented in Fig. 1. The phosphorylation of mitogen-activated protein kinases (MAPKs) and the production of reactive oxygen species (ROS) both represent activation of signal transduction cascades, and are frequently used as immune signaling outputs, whereas nuclear calcium oscillations served as a proxy for responses associated with symbiosis. Using purified COs, Zhang et al. (9) showed that CO8 activated both symbiosis-associated nuclear calcium oscillations alongside MAPK and ROS activation, whereas CO4 only elicited nuclear calcium oscillations. These data suggest that early perception of AMF by plants most likely involves CO8 and possibly also CO4, confirming reports by others (10, 11).

The observation that pretreatment with CO4 suppressed CO8-induced ROS, and the confirmation that this suppression requires the CO4-binding receptor, Myc Factor Receptor1, also previously named Nod Factor Receptor5 (MYR1/NFR5) (12, 13), hinted at the possibility of complex receptor dynamics at the plasma membrane. A series of biochemical experiments performed in *Arabidopsis thaliana* protoplasts demonstrated that CO4 attenuated CERK1–CEBiP interaction, and this was further enhanced by the presence of MYR1. Consistent with the biochemical evidence, the physiological phenotypes of *myr1* mutants included higher levels of CO8-elicited ROS, increased expression of defense genes, and reduced colonization by the rice blast fungus *Magnaporthe oryzae*. These assays together support the notion that, in the absence of MYR1, the CERK1–CEBiP immune response is stronger.

The fine balance between mutualism and immunity was further demonstrated by the enhanced susceptibility of rice to the blast fungus in plants overexpressing *MYR1-GFP*. As demonstrated in this and earlier work (9, 11), upon perception of AMF, both immunity and symbiosis signaling responses are activated—therefore, loss of the immune receptor *CEBiP* is expected to increase symbiosis signaling exerted by the CERK1–MYR1 complex. Accordingly, Zhang et al. noted a subtle increase in mycorrhizal symbiosis in *cebip* mutants, which occurred at early stages of the symbiosis (2 wk postinoculation) and was lost thereafter (6, 9). As it could have been anticipated that there is a continuous need for controlled immunity signaling throughout symbiosis establishment, the modest and transient promotion of symbiosis in the absence of immunity-activating CERK1–CEBiP complex is an enigma for further investigation.

Taken together, the work by Zhang et al. provides evidence for a model in which the CERK1–CEBiP complex underpins immune responses whereas the CERK1–MYR1/NFR5 complex activates symbiosis signaling in the presence of beneficial AMF. Competition between the receptor complexes thus results in the antagonistic signaling outputs of symbiosis and immunity—as summarized in Fig. 1.

There are, however, many outstanding questions. For instance, how are the diverse chitinaceous signals—CO4, CO8, and LCOs—integrated upon perception by a plant? We now appreciate that a broad range of fungi (including Basidiomycetes and Ascomycetes) produce LCOs (14), a compound classically regarded as a symbiotic signal based on its prior identification as rhizobial nod factors. Chitin, on the other hand, is an indispensable component of fungal cell walls and substrate for abundant plant chitinases, whose activity yields COs including CO4 and CO8. It thus seems that the rhizosphere is inevitably abundant in CO8, CO4, and LCOs. Both CO4 and LCOs are capable of attenuating the early immunity-associated ROS and MAPK activation that CO8 elicits (11, 15). All of these chitin signals activate nuclear calcium oscillations (a hallmark of symbiosis signaling), but CO8 ultimately suppressed symbiosis with AMF in this work (9). Zhang et al. (9) focus their work on CO4, mentioning that LCOs are not perceived by rice, although it is worth noting that the authors previously demonstrated occurrence of nuclear calcium oscillations at higher concentrations of LCOs in rice (13), consistent with observations made in other plant species (16).

Another important unanswered question relates to the known ligand promiscuity of the receptors, not strictly binding either to CO4 or CO8 (13, 17), and how this can be factored into the model. This and previous work have demonstrated that CEBiP binds to CO4 and CO8 with similar affinities (17), whereas MYR1 binds to CO4 and at a lower affinity to CO8 (13). What then, is the relative population of CO4- and CO8-bound receptor complexes and their composition? Clearly, more work will be needed to understand how exactly plants use CO8, CO4, and LCO perception to distinguish beneficial and pathogenic microbes. Receptor competition presented in this manuscript offers one mechanism that marks the beginning toward a more complete understanding on how the plant kingdom perceive the second-most abundant polymer in terrestrial landscapes, and how plants form symbioses with the appropriate microorganisms.
